# Supplementary material for: Comparative genomics provides new insights into the diversity, physiology, and sexuality of the only industrially exploited tremellomycete: Phaffia rhodozyma
Source: BMC Genomics. 2016 Nov 9;17:901. doi: 10.1186/s12864-016-3244-7 (PMC5103461; doi:10.1186/s12864-016-3244-7)
Supplement: Additional file 6: — List of orphan genes with links to PFAM (related to Additional file 1: Table S1). (ZIP 1428 kb) [file 12864_2016_3244_MOESM6_ESM.zip › BLAST_HTML_FTR/G01194_P.html]

BLAST Search Results


```
BLASTP 2.2.27+


Reference:
Stephen F. Altschul, Thomas L. Madden, Alejandro A. Schäffer,
Jinghui Zhang, Zheng Zhang, Webb Miller, and David J. Lipman (1997),
"Gapped BLAST and PSI-BLAST: a new generation of protein database
search programs", Nucleic Acids Res. 25:3389-3402.


Reference for
composition-based statistics:
Alejandro A. Schäffer, L. Aravind, Thomas L. Madden, Sergei
Shavirin, John L. Spouge, Yuri I. Wolf, Eugene V. Koonin, and
Stephen F. Altschul (2001), "Improving the accuracy of PSI-BLAST
protein database searches with composition-based statistics and
other refinements", Nucleic Acids Res. 29:2994-3005.


Database: nr
           71,551,133 sequences; 26,053,659,533 total letters


Query= G01194_P

Length=562
                                                                      Score     E
Sequences producing significant alignments:                          (Bits)  Value

emb|CED82909.1|  hypothetical protein [Xanthophyllomyces dendrorh...  1103    0.0  
ref|XP_011528930.1|  PREDICTED: uncharacterized protein LOC101927...  45.1    0.24 
gb|EUC66792.1|  hypothetical protein RSOL_509150 [Rhizoctonia sol...  43.1    0.85 
gb|KEP53177.1|  hypothetical protein V565_034750 [Rhizoctonia sol...  43.1    0.94 


 >emb|CED82909.1| hypothetical protein [Xanthophyllomyces dendrorhous]
Length=561

 Score = 1103 bits (2852),  Expect = 0.0, Method: Compositional matrix adjust.
 Identities = 561/561 (100%), Positives = 561/561 (100%), Gaps = 0/561 (0%)

Query  1    MIALGNPRTTTRLEPSSMPFRPHPLTPKRPAGQSQPFFPSSFLPALQEDPQSRALDGWLV  60
            MIALGNPRTTTRLEPSSMPFRPHPLTPKRPAGQSQPFFPSSFLPALQEDPQSRALDGWLV
Sbjct  1    MIALGNPRTTTRLEPSSMPFRPHPLTPKRPAGQSQPFFPSSFLPALQEDPQSRALDGWLV  60

Query  61   DGDELSSLAAQSDTHRSVHLVLGDPSAESLDLILDQLEANTLLLLPPLASLSTEAVTLLK  120
            DGDELSSLAAQSDTHRSVHLVLGDPSAESLDLILDQLEANTLLLLPPLASLSTEAVTLLK
Sbjct  61   DGDELSSLAAQSDTHRSVHLVLGDPSAESLDLILDQLEANTLLLLPPLASLSTEAVTLLK  120

Query  121  RQPRQHRSQRSLVRHLDEALTSPTAISALLTQAAEYAVRWKLPSTRKTSARLGMMIPSAK  180
            RQPRQHRSQRSLVRHLDEALTSPTAISALLTQAAEYAVRWKLPSTRKTSARLGMMIPSAK
Sbjct  121  RQPRQHRSQRSLVRHLDEALTSPTAISALLTQAAEYAVRWKLPSTRKTSARLGMMIPSAK  180

Query  181  QKSRQDRDQDDQSQQPFSPQTDPTLDSVVYFLPPPTDNSLLKEQLSLAFLITTASISLLS  240
            QKSRQDRDQDDQSQQPFSPQTDPTLDSVVYFLPPPTDNSLLKEQLSLAFLITTASISLLS
Sbjct  181  QKSRQDRDQDDQSQQPFSPQTDPTLDSVVYFLPPPTDNSLLKEQLSLAFLITTASISLLS  240

Query  241  PKAFRRQTTTRSNLSTHRGSFSTPSPTPRSRSTFLSSDPSPSIDRSDSATASKVKSGSFV  300
            PKAFRRQTTTRSNLSTHRGSFSTPSPTPRSRSTFLSSDPSPSIDRSDSATASKVKSGSFV
Sbjct  241  PKAFRRQTTTRSNLSTHRGSFSTPSPTPRSRSTFLSSDPSPSIDRSDSATASKVKSGSFV  300

Query  301  SFSEAGSSRSATSTPTPSTGPARMGMLVHILPTLARSPSSDGTFHAVFARSLDKFQQRFL  360
            SFSEAGSSRSATSTPTPSTGPARMGMLVHILPTLARSPSSDGTFHAVFARSLDKFQQRFL
Sbjct  301  SFSEAGSSRSATSTPTPSTGPARMGMLVHILPTLARSPSSDGTFHAVFARSLDKFQQRFL  360

Query  361  EKNLAITMVTLPEEILQDSPVPAPDGLGRSSSWSSRLSIPEEEEESPKDIPPSPPMTTSV  420
            EKNLAITMVTLPEEILQDSPVPAPDGLGRSSSWSSRLSIPEEEEESPKDIPPSPPMTTSV
Sbjct  361  EKNLAITMVTLPEEILQDSPVPAPDGLGRSSSWSSRLSIPEEEEESPKDIPPSPPMTTSV  420

Query  421  VTLTNTHNVISLIFSSAIKSPLPPLPSRSRRGSSSSLASRTSFQSISQYGSGPGLGYPAL  480
            VTLTNTHNVISLIFSSAIKSPLPPLPSRSRRGSSSSLASRTSFQSISQYGSGPGLGYPAL
Sbjct  421  VTLTNTHNVISLIFSSAIKSPLPPLPSRSRRGSSSSLASRTSFQSISQYGSGPGLGYPAL  480

Query  481  ADVNSFLPSADAIMFIPSSTGSLTSSSASSAYSDHKSRHQADLITQTTFPSRPTSSIINL  540
            ADVNSFLPSADAIMFIPSSTGSLTSSSASSAYSDHKSRHQADLITQTTFPSRPTSSIINL
Sbjct  481  ADVNSFLPSADAIMFIPSSTGSLTSSSASSAYSDHKSRHQADLITQTTFPSRPTSSIINL  540

Query  541  SRKQEKNGWIGRLFAKRERGC  561
            SRKQEKNGWIGRLFAKRERGC
Sbjct  541  SRKQEKNGWIGRLFAKRERGC  561


>ref|XP_011528930.1| PREDICTED: uncharacterized protein LOC101927859 isoform X5 [Homo 
sapiens]
Length=1974

 Score = 45.1 bits (105),  Expect = 0.24, Method: Composition-based stats.
 Identities = 111/465 (24%), Positives = 173/465 (37%), Gaps = 56/465 (12%)

Query  77    SVHLVLGDPSAESLDLILDQLEANTLLLLPPLASLSTEAVTLLKRQPRQHRSQR-----S  131
             +VH +  + +  S   +   L   +L  + P ASL T  +  L R+P +    R     S
Sbjct  1426  AVHSIAIEDTTPSRAWLTRSLPRASLPRMSPTASLRTPPMASLTRRPPRPSLSRMPPTAS  1485

Query  132   LVRHLDEALTSPTAISALLTQAAEYAVRWKLP---STRKTSARLGMMIPSAKQKSRQDRD  188
             L R    A  + T  +A LT+    A   + P   S R+T +R        +    +   
Sbjct  1486  LTRMPSRASLTRTPPTASLTRTPPRASLTRSPPMASLRRTPSRASRTRTPPRASRTRTPP  1545

Query  189   QDDQSQQPFSPQTDPTLDSVVYFLPPPTDNSLLKEQLSLAFLITTASISLLSPKAFRRQT  248
             +   ++ P       T  +      PPT         SL  +  T S+S   P A   ++
Sbjct  1546  RASHTRTPSRALLTRTPSTASLKRSPPT--------ASLTRMPPTRSLSRTPPAASLTRS  1597

Query  249   TTRSNLSTHRGSFSTPSPTPRSRSTFLSSDPSPSIDRSDSAT--------ASKVKSGSFV  300
               R++L     + S     P +  T   S P+ S+ RS S          AS  +S    
Sbjct  1598  PPRASLPRMSPTASLTRTPPTASLT--RSPPTASLTRSPSTASLSRTPPRASLKRSPPTA  1655

Query  301   SFSEAGSSRSATSTPTPSTGPARMGMLVHI-----LPTLARSPSSDGTFHAVFARSLDKF  355
             S +   S  S TSTP+ ++   RM     +     + +L RSP +     A   R+L + 
Sbjct  1656  SLTRTPSRASLTSTPSTASSLTRMPPKQSLSRTPPMASLTRSPPT-----ASRTRTLPR-  1709

Query  356   QQRFLEKNLAITMVTLPEEILQDSPVPAPDGLGRSSSWSSRLSIPEEEEESPKDIPPSPP  415
                      A    T P   L  SP  A   L R+ S +SR   P     +      S  
Sbjct  1710  ---------ASRTRTPPRASLTRSPPTA--SLRRTPSRASRTRTPPRASRTRTPPRASHT  1758

Query  416   MTTSVVTLTNTHNVISLIFSSAIKSPLPPLPSRSRRGSSSSLASRTSFQSISQYGS-GPG  474
              T S   LT T +  SL  S       PP  S +R  S +SL    S  S+++  S    
Sbjct  1759  RTPSRALLTRTPSTASLKRS-------PPTASLTRTPSRASLTRTPSRASLTRLKSRASH  1811

Query  475   LGYPALADVNSFLPSADAIMFIPSSTGSLTSSSASSAYSDHKSRH  519
                P+ A +    P+A      P+++ + T   AS   +  ++ H
Sbjct  1812  TRTPSRASLTRTPPTASLTRSPPTASRTRTPPRASRTRTPPRASH  1856


>gb|EUC66792.1| hypothetical protein RSOL_509150 [Rhizoctonia solani AG-3 Rhs1AP]
Length=657

 Score = 43.1 bits (100),  Expect = 0.85, Method: Compositional matrix adjust.
 Identities = 93/374 (25%), Positives = 142/374 (38%), Gaps = 78/374 (21%)

Query  19   PFRPHPLTPKRPAGQSQPFFPSSFLPALQEDPQSRALDGWLVDGDELSSLAAQSDTHRSV  78
            PFRP    P  P  QSQ   P++        P  RA   W +D   L +L    +  RSV
Sbjct  58   PFRPASQYPADPYAQSQSKRPTT---ERSSRPHRRA--PWTLD---LEALEGLQEPQRSV  109

Query  79   HLVLGDPSAESLDLILD-QLEANTLLLLPPL-------ASLSTEAVTLLKRQPRQHRSQR  130
             L+LGDPS  +L  +L  +  A++LL++P          + ST  + L+   P     Q 
Sbjct  110  ILILGDPSPLALKPLLGIKSIASSLLIVPSTRPSLDVPQNESTPEIRLVPIDPES--PQE  167

Query  131  SLVRHLDEALTSPTAISALLTQAAEYAVRWKLPSTRKTSARLGMMIPSAKQKSRQDRDQD  190
               R  D           ++  A + A +W+           G +     Q+SR      
Sbjct  168  GAARVAD-----------VMKSAEKIARQWRREMADAEQPASGTLSRPPTQRSRLSVMHS  216

Query  191  DQSQ---QPFSPQTDPT------------------LDSVVYFLPPPTDNSLLKEQLSLAF  229
            + +Q    P +P+  P                   LD V+ FLP      ++KE + +  
Sbjct  217  NNAQLLTPPTTPRIHPVSRLPKSRDRQSYINQQRALDMVINFLPNANAKGIIKEAILVTT  276

Query  230  LITTASISLLSPKAFRRQTTTRSNLSTHRGSFSTPSPTPRSRSTFLSSDPSPSIDRSDSA  289
            ++  +   L  PK+               G FS+    P      LS  PS ++D   ++
Sbjct  277  IVKPSLFPLSPPKS---------------GMFSSGVTEPP-----LSRVPS-TVDLMGTS  315

Query  290  TASKVKSGS----FVSFSEAGSSRSATSTPTPSTGPARMGMLVHILPTLARSPSSDGTFH  345
              S++ SGS     +S S   S   A+ +P     PAR   ++HILP     P      H
Sbjct  316  RWSRLFSGSRTSLVISNSNPRSQSPASLSPPAPASPAR---IIHILPFTYLPPPQGPFAH  372

Query  346  AVFARSLDKFQQRF  359
            A   R L+ F   F
Sbjct  373  AHLIRKLEAFLLDF  386


>gb|KEP53177.1| hypothetical protein V565_034750 [Rhizoctonia solani 123E]
Length=652

 Score = 43.1 bits (100),  Expect = 0.94, Method: Compositional matrix adjust.
 Identities = 93/374 (25%), Positives = 142/374 (38%), Gaps = 78/374 (21%)

Query  19   PFRPHPLTPKRPAGQSQPFFPSSFLPALQEDPQSRALDGWLVDGDELSSLAAQSDTHRSV  78
            PFRP    P  P  QSQ   P++        P  RA   W +D   L +L    +  RSV
Sbjct  58   PFRPASQYPADPYAQSQSKRPTT---ERSSRPHRRA--PWTLD---LEALEGLQEPQRSV  109

Query  79   HLVLGDPSAESLDLILD-QLEANTLLLLPPL-------ASLSTEAVTLLKRQPRQHRSQR  130
             L+LGDPS  +L  +L  +  A++LL++P          + ST  + L+   P     Q 
Sbjct  110  ILILGDPSPLALKPLLGIKSIASSLLIVPSTRPSLDVPQNESTPEIRLVPIDPES--PQE  167

Query  131  SLVRHLDEALTSPTAISALLTQAAEYAVRWKLPSTRKTSARLGMMIPSAKQKSRQDRDQD  190
               R  D           ++  A + A +W+           G +     Q+SR      
Sbjct  168  GAARVAD-----------VMKSAEKIARQWRREMADAEQPASGTLSRPPTQRSRLSVMHS  216

Query  191  DQSQ---QPFSPQTDPT------------------LDSVVYFLPPPTDNSLLKEQLSLAF  229
            + +Q    P +P+  P                   LD V+ FLP      ++KE + +  
Sbjct  217  NNAQLLTPPTTPRIHPVSRLPKSRDRQSYINQQRALDMVINFLPNANAKGIIKEAILVTT  276

Query  230  LITTASISLLSPKAFRRQTTTRSNLSTHRGSFSTPSPTPRSRSTFLSSDPSPSIDRSDSA  289
            ++  +   L  PK+               G FS+    P      LS  PS ++D   ++
Sbjct  277  IVKPSLFPLSPPKS---------------GMFSSGVTEPP-----LSRVPS-TVDLMGTS  315

Query  290  TASKVKSGS----FVSFSEAGSSRSATSTPTPSTGPARMGMLVHILPTLARSPSSDGTFH  345
              S++ SGS     +S S   S   A+ +P     PAR   ++HILP     P      H
Sbjct  316  RWSRLFSGSRTSLVISNSNPRSQSPASLSPPAPASPAR---IIHILPFTYLPPPQGPFAH  372

Query  346  AVFARSLDKFQQRF  359
            A   R L+ F   F
Sbjct  373  AHLIRKLEAFLLDF  386


Lambda      K        H        a         alpha
   0.313    0.127    0.358    0.792     4.96 

Gapped
Lambda      K        H        a         alpha    sigma
   0.267   0.0410    0.140     1.90     42.6     43.6 

Effective search space used: 6046023210710


  Database: nr
    Posted date:  Sep 23, 2015 12:05 AM
  Number of letters in database: 26,053,659,533
  Number of sequences in database:  71,551,133


Matrix: BLOSUM62
Gap Penalties: Existence: 11, Extension: 1
Neighboring words threshold: 11
Window for multiple hits: 40
```
